# A Cross-Sectional Multicenter Study of Workplace Violence against Prehospital Emergency Medical Technicians

**DOI:** 10.1155/2018/7835676

**Published:** 2018-04-04

**Authors:** Seyed Hamid Hosseinikia, Shekufeh Zarei, Majid Najafi Kalyani, Sepideh Tahamtan

**Affiliations:** ^1^Student Research Committee, Fasa University of Medical Sciences, Fasa, Iran; ^2^Fasa University of Medical Sciences, Fasa, Iran; ^3^School of Nursing and Midwifery, Shiraz University of Medical Sciences, Shiraz, Iran

## Abstract

**Background and Purpose:**

Workplace violence is a global phenomenon and violation of human rights affects the people's self-esteem and quality of work and causes inequality, discrimination, disorder, and conflict at work. The present study was carried out aiming at determining the workplace violence against the prehospital emergency medical technicians (PEMTs) in three provinces of Fars, Kohgiluyeh and Boyer-Ahmad, and Bushehr, Iran.

**Materials and Methods:**

This was a cross-sectional multicenter study in which 206 PEMTs from Fars, Bushehr, and Kohgiluyeh and Boyer-Ahmad provinces participated. Simple random sampling was used in this study. In order to collect data, a researcher-made tool was used. Descriptive statistics and SPSS® software version 22 were used to analyze the data.

**Results:**

Among various types of workplace violence, the most frequent ones were verbal violence (78.1%), physical violence (60.3%), and cultural violence (31.7%), respectively. The most important factor in the occurrence of workplace violence was the lack of the awareness of people about the duties of the PEMTs. With regard to the handling of the violent situations, the results indicated that 61.6% of the personnel asked the attacker to calm down. 48.5% of PEMTs believed that violence was normal in their work.

**Conclusion:**

Due to the high rate of workplace violence against PEMTs, it is suggested that methods such as formal training and retraining programs for the employees, general education with regard to the duties of the PEMTs, and socially supporting them should be used to reduce and control violence.

## 1. Introduction

Workplace violence is defined as any incident or situation in which a person is abused, threatened, or attacked at the workplace and/or conditions related to it [1]. Workplace violence affects the people's self-esteem and quality of work and causes inequality, discrimination, disorder, and conflict at work [2].

Despite the fact that violence occurs in all work environments, health workers are more exposed to workplace violence [3]. Statistics show that annually 70–80% of physicians, nurses, emergency medical personnel, and public service staff experience one or more cases of violence [4].

With regard to the nature of the work of the PEMTs, they are always at risk of confronting violence by the patient or their companions. PEMTs often encounter patients in critical situations that require immediate medical attention. In such stressful cases, they must provide emergency services and medical first aid to the patients at the scene of the accident and on the way back to health centers, and they should transfer the patients to hospitals and other health centers for performing specialized cares as soon as possible [5]. The results of a previous study showed that 83.3% of the employees had experienced workplace violence at least once a year [6].

The rate of workplace violence is constantly increasing, the reason of which is the absence of preventive strategies and effective reporting system. The reports resulting from a survey that examined the extent of exposure to violence among emergency medical personnel showed that 25% of the personnel were exposed to physical attacks and physical injuries [7, 8]. The results of a study conducted by Gormley et al. on violence against US emergency medical personnel showed that 69% of the personnel experienced various kinds of violence over the previous year, and the rate of verbal violence had been more than any other physical violence [9].

A survey from the emergency medical personnel in two Canadian provinces showed that 75% of the respondents had experienced violence in the past year [10]. Verbal violence is the most common type of violence reported by the emergency medical personnel [11]. In a study, Maguire et al. showed that work-related harms among the emergency medical personnel were three times higher than the global average for other occupations [12].

Unfortunately, acts of violence cause many physical and psychological complications, such as physical injuries, tension headaches, anger, fear, depression, anxiety, feeling of guilt, decreased self-esteem, undesirable effects on the quality of patient care, reduced work morals, occupational burnout, frustration, and negative attitudes towards work. Additionally, disturbances such as the loss of working days, constraints on activity or work, termination of employment, job change, and even death are serious complications of occupational violence [13–16]. Research shows that there is a relationship between the incidence of occupational accidents and absenteeism and this relationship has a negative impact on the employees' job satisfaction. In addition, violence can affect the whole healthcare team and affect the quality of services provided to patients [17, 18].

Most cases of violence in the hospitals have been caused by patients, patient's relatives, physicians, personnel working in the hospital, and visitors [19, 20]. Unfortunately, despite the great importance of workplace violence and its destructive effects, this problem has not yet been taken seriously in our country's healthcare system, and various studies have examined this problem only in a single province and in a small area. Therefore, with regard to the importance of this issue, as well as the lack of research performed on PEMTs, the present study was conducted aiming to investigate the workplace violence against PEMTs in Fars, Kohgiluyeh and Boyer-Ahmad, and Bushehr provinces, Iran.

## 2. Materials and Methods

### 2.1. Settings and Participants

A total of 206 PEMTs working in the prehospital emergency centers (city-road) in Fars, Kohgiluyeh and Boyer-Ahmad, and Bushehr provinces were selected through simple random sampling and enrolled in the study. The criteria for inclusion in the study were having a bachelor's degree in nursing or an associate degree or bachelor's degree in prehospital emergency nursing, at least one year of work experience in a prehospital emergency center, and willingness to participate in the study; the criterion for exclusion from the study was the incomplete filling out of the questionnaires.

### 2.2. Method

This study was a cross-sectional multicenter study conducted between August 2016 and 2017. In this research, a researcher-made questionnaire was used to determine the prevalence of workplace violence against the emergency medical personnel and the factors influencing the occurrence of this type of violence.

### 2.3. Ethical Considerations

The regional ethics committee of Fasa University of Medical Sciences approved the study. After the approval of the ethics committee and obtaining informed consent, the questionnaires were distributed among the PEMTs.

### 2.4. Instruments

The questionnaire used in this study was developed after extensive examination of the texts and by combining the existing questionnaires. The validity of the questionnaire was confirmed by using the viewpoints of the experts and the faculty members of Fasa University of Medical Sciences. To determine the reliability of the questionnaire, before carrying out the research, the above-mentioned questionnaire was distributed randomly among a population similar to the research population, 30 members of the PEMTs, and the internal correlation of the items was calculated to be equal to 0.85, using Cronbach's alpha test. The questionnaire has four sections. The first section is about the demographic characteristics of the personnel including age, level of education, type of employment, work experience, number of assignments per week, and prior training in anger management. Section two examines different types of workplace violence experienced including physical, verbal, and cultural violence, which includes the experience of violence, the frequency of violence, the attacker, and the place of the occurrence of violence. Cultural violence in this questionnaire refers to any act regarding ridiculing the language, culture, appearance, and accent of PEMTs. In the third section, the response of the personnel to the workplace violence and the reason for this response and in the fourth section the factors influencing the act of violence were investigated.

### 2.5. Data Analysis

The questionnaires that had not been filled up completely were removed from the analysis process. Data analysis was performed through SPSS software version 22, using descriptive statistical tests.

## 3. Results

All participants in this study were male. The mean age of the subjects was 31.77 ± 6.24 years and their age range was 22 to 53 years. 48 (23.6%) of these employees were official or contractual employees, and the rest were part-time and medical service plan employees. 61.4% of the personnel had an associate degree and the rest had a bachelor's degree. The mean work experience of the individuals was 7.84  ±  5.43 years and the mean of the number of assignments per week was 16.65 ± 16.89. One hundred fifty-three (74.3%) individuals reported that they had not received any prior training in the field of anger management.

The findings of the study showed that, among different types of workplace violence, the most frequent types were verbal violence (78.1%), physical violence (39.3%), and cultural violence (31.1%), respectively. The results also showed that the majority of the people responsible for violence in the verbal abuse, physical violence, and cultural violence were the people present at the scene (49.4%), the relatives of the patient (48.2%), and the people present at the scene (47%), respectively. Regarding the factors causing workplace violence from the viewpoint of PEMTs, the findings showed that the most important factor in the incidence of violence was the lack of awareness of the duties of PEMTs (Table 1).

As to the reaction of the PEMTs towards violence, the results showed that 61.6% of the personnel asked the invading individual to stay calm (Table 2). 48.5% of the PEMTs believed that violence was normal in their work (Table 3).

## 4. Discussion

The present study was conducted to investigate workplace violence against PEMTs in Fars, Kohgiluyeh and Boyer-Ahmad, and Bushehr provinces, Iran. Workplace violence is a multidimensional issue, and many factors contribute to its incidence. Therefore, the rate of the incidence of workplace violence in healthcare or prehospital centers can vary from one country to another and even from one city to another due to cultural differences, special characteristics of health centers, different occupational nature of the job of the medical personnel, and the behavioral characteristics of healthcare personnel [21].

The findings of this study showed that the highest rate of violence against PEMTs was related to verbal violence. The results of this study are consistent with similar studies. The prevalence of verbal violence in other similar studies is reported to be between 20% and 90% [10]. The results of a study carried out by Alhardy in 2017 on the workplace violence against the emergency medical personnel showed that the highest rate of violence was the verbal violence (61%) [22]. The results of the research by Gormley et al. in 2015 showed that, among emergency medical personnel, verbal violence and physical violence were the most frequent cases of violence against personnel with 67% and 43.6%, respectively [9]. In the study of Pourshaikhian et al., the frequency of verbal violence, physical violence, and cultural violence was reported as 21 to 82%, 13–79%, and 9.5%, respectively [4].

The results of this study showed that among the parties responsible for violence the relatives of the patients and people present at the scene were the majority of the cases. In the study of Alhardy, it was determined that the majority of the parties responsible for violence were the attendants of the patient (80%) [22]. Also, in the study by Koohestani et al., most of the cases of violence were done by the attendants of the patients [21]. These results are consistent with that obtained in the current study.

In this study, the lack of awareness about the duties of PEMTs was reported as the most important effective factor in the incidence of violence from the viewpoint of the personnel. Many people do not have a correct image of the duties of PEMTs and in many occasions mistake the personnel with physicians and expect an appropriate treatment and the prescription of various medications for their patient, and when this expectation is not met, they show violent behavior [23].

The most important reaction of the PEMTs to violence was asking the invading individuals to stay calm. Emergency medical employees expect violence from their patient and their relatives through their experiences, and also the personnel prefer not to show any reaction and try to ask the attacking individual to stay calm as a result of attending training courses at the beginning of their service and courses teaching them how to communicate with patients and their attendants [6]. The results of the study by Rahmani et al. also confirmed this to be the case and showed that calling the violent party to calm down was the response of the majority of participants in the study [23].

Healthcare personnel should avoid any violent reactions similar to that of the patient and his or her attendant when facing violence from the patients and their attendants; their responsibility is to calm the patient and his relatives. In cases where the healthcare personnel encounter a violent situation caused by the patient or his/her relatives, they should first evaluate the condition of the attacker and the harming individual and avoid agitating him/her as much as possible and try to ask him/her to be quiet and calm. As a matter of fact, the personnel exposed to violence can make the most appropriate decision in critical moments by controlling their behavior and nervous tension, reduce the severity of the violence of the patient and his/her attendants, and reduce the extent of the incidental damage [21].

The results of this study showed that most of the PEMTs believe violence is normal in their job and believe that following-up violent incidents is useless. The results of other studies showed that only 10% of the personnel reported the incidence of violence to higher authorities. These people considered the main reasons for not reporting was that reporting was useless or this violence was not important [22].

Also, in the study of Heydarikhayat et al., 22.5% believed that reporting violence was futile and causes trouble and did not help to change the current situation and prevent occupational violence; therefore, they refused to report the violence [24]. The study of Rahmani et al. also showed that the emergency personnel considered following-up workplace violence useless (34%) and regarded violence as normal in their work (30%). In addition, the participating personnel in the study conducted by Rahmani et al. mentioned that they did not know to whom they should report the violence (13%) [25].

Considering the results of the present study and the high prevalence of workplace violence among PEMTs, it is suggested that strategies should be implemented to reduce workplace violence. Developing instructions and formal training of the personnel, retraining programs for reducing or controlling workplace violence against PEMTs, passing and enforcing laws necessary for safety during emergency cases and protecting the personnel, general public education in relation to the duties of PEMTs, and socially supporting them are some of the measures that can be taken with regard to the violence against healthcare personnel [4]. In the meantime, training is one of the best strategies to reduce workplace violence against the PEMTs. The first level of training is to educate the public about the performance and duties of the PEMTs. The second level of training is training the PEMTs in the field of the management of violence. Considering the special occupational conditions, a complete control of violence in the prehospital emergency is a bit difficult; however, the skill of the PEMTs in controlling violence is also a very important factor [23].

One of the limitations of this study was failing to examine other factors related to the incidence of workplace violence against the PEMTs, where the viewpoint of the managers and authorities of the universities and the disaster and emergency medical centers and also the factors related to the patients and the relatives of the patients can be some of these factors. Accordingly, conducting qualitative research in this regard is suggested.

## Figures and Tables

**Table 1:** Factors affecting occupational violence from the perspective of PEMTs.

Associated factors	*N*	%
Drug abuse	4	1.9
Patient's death	12	5.8
Lack of awareness of people about the duties of emergency medical personnel	111	53.9
Delay in arriving at the accident site	14	6.8
The lack of retraining programs for personnel in the field of violence management	8	3.9
Shortage in ambulance personnel	56	27.2
Shortage of security equipment	1	0.5

**Table 2:** Types of the reactions of PEMTs to workplace violence.

Type of reaction	*N*	%
I showed no reaction	53	25.7
I asked the aggressor to stay calm	127	61.6
I consulted with my colleagues	2	1
I defended myself	15	7.3
I reported the incident to the management	5	2.4
Escape	4	1.9

**Table 3:** The reasons for the reaction of PEMTs to workplace violence.

The reason	*N*	%
Violence is a normal thing in our work	100	48.5
It was my fault	1	0.5
Following-up violent incidents is useless	95	46.1
I did not know who to report to	10	4.9
